# A Brief History of IL-1 and IL-1 Ra in Rheumatology

**DOI:** 10.3389/fphar.2017.00293

**Published:** 2017-05-23

**Authors:** Jean-Michel Dayer, Francesca Oliviero, Leonardo Punzi

**Affiliations:** ^1^Faculty of Medicine, University of GenevaGeneva, Switzerland; ^2^Department of Medicine, University of PadovaPadova, Italy

**Keywords:** interleulin-1, interleukin-1 antagonist, rheumatoid arthritis, inflammasome, autoinflammatory diseases

## Abstract

The history of what, in 1979, was called interleukin-1 (IL-1), orchestrator of leukocyte inter-communication, began many years before then, initially by the observation of fever induction via the endogenous pyrogen (EP) (1974) and then in rheumatology on the role in tissue destruction in rheumatoid diseases via the induction of collagenase and PGE_2_ in human synovial cells by a mononuclear cell factor (MCF) (1977). Since then, the family has exploded to presently 11 members as well as many membrane-bound and soluble receptor forms. The discovery of a natural Interleukin-1 receptor antagonist (IL-1Ra) in human biological fluids has highlighted the importance of IL-1 and IL-1Ra in human diseases. Evidence delineating its role in autoinflammatory syndromes and the elucidation of the macromolecular complex referred to as “inflammasome” have been instrumental to our understanding of the link with IL-1. At present, the IL-1blockade as therapeutic approach is crucial for many hereditary autoinflammatory diseases, as well as for adult-onset Still’s disease, crystal-induced arthropathies, certain skin diseases including neutrophil-triggered skin diseases, Behçet’s disease and deficiency of IL-1Ra and other rare fever syndromes. Its role is only marginally important in rheumatoid arthritis and is still under debate with regard to osteoarthritis, type 2 diabetes mellitus, cardiovascular diseases and cancer. This brief historical review focuses on some aspects of IL-1, mainly IL-1β and IL-Ra, in rheumatology. There are many excellent reviews focusing on the IL-1 family in general or with regard to specific diseases or biological discoveries.

## IL-1 and Its Roots

Historically in rheumatology, the seminal observation of the production of human interstitial collagenase (later called MMP-1) by human synovial cells in patients with rheumatoid arthritis (RA) led to the unraveling of the link between matrix degradation and the biological function of inflammatory molecules produced by immune cells. It was found that the main cellular source of both interstitial collagenase (matrix metalloproteases, MMPs) and prostaglandin E_2_ (PGE_2_) in synovial tissue were adherent stellate fibroblast-like cells (ASC), also called type B fibroblastic synovial lining cells (Dayer et al., 1976). Presently, the fibroblast subpopulation in inflamed synovial tissue has proved to be far more complex (Croft et al., 2016).

**FIGURE 1:**
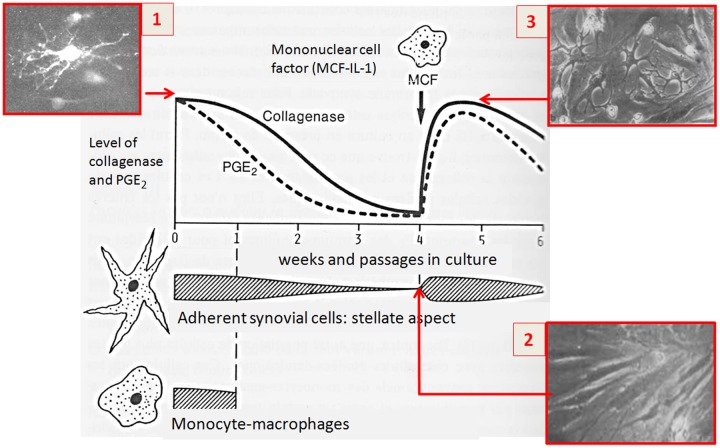
**Seminal observation about the link between matrix degradation and the biological function of inflammatory molecules produced by immune cells (1976–1977).** (1) Cultures of synovial explants from patients with rheumatoid arthritis release large quantities of collagenase and prostaglandins (PGE2) into the media. Secretion of PGE2 declines more rapidly with respect to collagenase whose levels decline after different passages in culture; (2,3) The addition of monocyte-macrophages or their culture supernatants restored collagenase and PGE2 levels. The soluble factor responsible of this effect was later identified in mononuclear cell factor (MCF).

The question was which are the driving forces for stimulating the synovial cells to produce MMP and PGE_2_. Seminal studies at the Arthritis Unit, Massachusetts General Hospital, Harvard Medical School) have shown that partial purification of the conditioned medium revealed a protein of approximately 15 kDa, and the factor responsible for this biological activity was called mononuclear cell factor (MCF) (Dayer et al., 1977a,b, 1981) (**Figure 1**). Interestingly, interactions between T cells and monocyte-macrophages were found to play an important role in the production of IL-1 by monocyte-macrophages, giving rise to the first descriptions of the pathways going from lymphocytes to monocyte-macrophages and synovial fibroblast cells (Dayer et al., 1979a,b).

At this early stage of the discovery, before the cloning, different investigators analyzed the soluble factors derived from leucocyte culture supernatants in their respective functional bioassays in order to identify the putative functional specificity. Purified ‘MCF’ proved to have similar properties to the lymphocyte-activating factor (LAF), a factor which had the same molecular weight of approximately 15 kDa and shared chromatographic as well as other biochemical properties (Mizel et al., 1981). It was not until 1979 that the nomenclature and the terminology of interleukin-1 (IL-1), orchestrator of leukocyte communication, was coined at a meeting (Ermatingen, Switzerland) and it referred to products previously identified by several investigators using different bioassays, including lymphocyte activating factor (LAF), MCF, B lymphocyte activating factor (BAFF) and of course endogenous pyrogen (EP) that induced fever. At that time, cytokines were defined only by biochemistry (Aarden et al., 1979). It was defined for the biochemistry properties by a m.w. between 12,000–18,000 with an isoelectric point of 4.5–5.5 in mouse and 6.5–7.5 in human, pH 2 insensitive, does not contain Ia and for the biologic properties as produced by macrophages, with H-2 unrestricted activity, unrestricted species activity, distinguishing bioactivity and does not possess the ability to promote and maintain *in vitro* long-term cultures of T cells in contrast to IL-2.

When human IL-1 was cloned using an antibody to EP and based on its EP bioactivity, the protein was expressed and the recombinant IL-1 found to have the same biological functions as MCF (Dayer et al., 1986). However, it must be emphasized that in addition to IL-1, natural tumor necrosis factor-cachectin (TNFα) was also demonstrated early on to induce MMP and PGE2 production in synovial cells (Dayer et al., 1985).

As far as LAF is concerned, despite the initial description of its role in the early activation of murine T cells from thymocytes, diseases characterized by an adaptive immune response to specific autoantigens are usually not part of the spectrum of IL-1β-driven disorders.

With regard to the field of rheumatology, other partially purified molecules like catabolin – inducing cartilage degradation – and osteoclast-activating factor (OAF) inducing calcium^45^ release from bone, to name but a few, were also found to have functions similar to those of IL-1 (Dayer, 2004).

The discovery of the other aspects of the IL-1 and IL-1 receptor families, related to the seminal features of EP and to several other important functions, has been extensively reviewed (Dinarello, 2010, 2015; Dinarello and van der Meer, 2013; Garlanda et al., 2013; Fantuzzi, 2016) as well as the biology of IL-1 alpha (Di Paolo and Shayakhmetov, 2016). The role of IL-1 in rheumatology and its specific interaction with MMPs have also been recently reviewed (Schett et al., 2016; Dayer et al., 2017).

## The Story Behind IL-1Ra

As early as 1984, before IL-1 was cloned, the existence of a potential natural inhibitor to IL-1 was suspected. In the bioassay for stimulating collagenase and prostaglandin production, no IL-1 biological activities were detected in the serum or urine of febrile patients (Balavoine et al., 1984). In the light of that finding, Dayer and colleagues (University of Geneva, Switzerland) reported in 1984 that, after biochemical purification, IL-1 was masked by a factor of approximately 17 kDa in the urine of febrile patients with monocytic leukemia and juvenile rheumatoid arthritis (JRA). This seminal observation was presented in 1984 at the Fourth International Lymphokine Workshop (Balavoine et al., 1984). The factor specifically blocked the biological activities of IL-1 (Dayer, 1985; Balavoine et al., 1986).

The first description of the concept underlying the interleukin-1 receptor antagonist (IL-1Ra) was that this natural antagonist could block the binding of a cytokine from the same family to its receptor without affecting those of TNFα (Seckinger et al., 1987a,b). In the field of cytokine, it was the first observation of an inhibitor factor that blocks ligand binding of cytokine and its activities. The discovery of the competitive binding assay of the IL-1 inhibitor (apparent m.w. around 17 kd) to IL-1 at the IL-1 receptor level was crucial for researchers at Synergen (Boulder, CO, United States) who purified and cloned IL-1Ra in 1990. Before IL-1Ra was cloned, the first clinical description of natural IL-Ra was made by following the time course of this specific IL inhibitor in the serum and urine of children with systemic juvenile chronic arthritis, a typical inflammatory syndrome (Prieur et al., 1987). Arend et al. (1985) reported the presence of an inhibitor of IL-1 activity under *in vitro* conditions, but neither its molecular weight nor the concept of binding to the IL-1 receptor was identified.

## IL-1 and Its Extended Family

First described as a secreted product of monocytes and neutrophils ∼35 years ago, interleukin (IL)-1 refers to IL-1α and IL-1β. At present, the IL-1 family comprises a total of 11 members, consisting of the two activating cytokines IL-1α and IL-1β, the IL-1Ra, as well as IL-18, IL-33, four isoforms of IL-36 [IL-36α, IL-36β, IL-36γ and IL-36 receptor antagonist (IL-36Ra)], IL-37 and IL-1 family member 10 (also known as IL-38). IL-1 is processed and activated by a caspase-1-dependent mechanism as well as by caspase-1-independent processes that involve neutrophil proteases (see history in Fantuzzi, 2016).

Interleukin-1 receptor antagonist, IL-36Ra and IL-37 are predominantly anti-inflammatory cytokines. IL-36Ra shares homology with IL-1Ra but is unable to bind to the IL-1R1 (Netea et al., 2015). It has an important role in regulating skin inflammation and its deficiency (linked to mutations in IL-36RN) is associated to a rare form of pustular psoriasis also called deficiency of IL-36-receptor antagonist or DITRA (Marrakchi et al., 2011). Skin lesions, along with multifocal osteomyelitis and periostitis are associated also to IL-1Ra deficiency, termed DIRA, an autosomal recessive autoinflammatory disease caused by mutations affecting IL1RN (Aksentijevich et al., 2009).

## Inflammasome, the Macromolecular Complex

**FIGURE 2:**
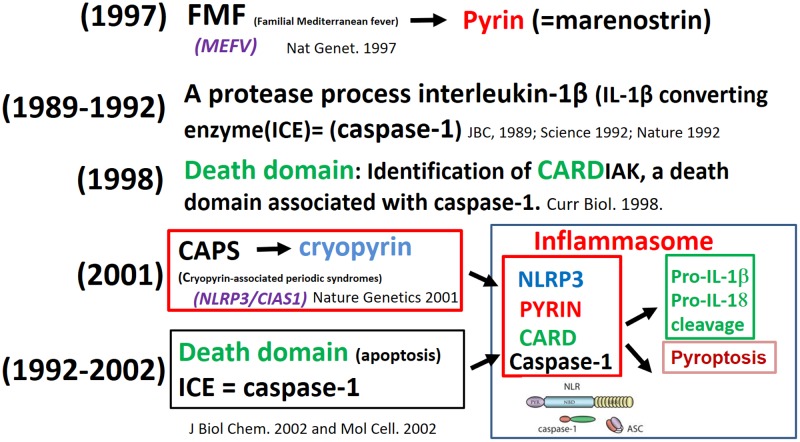
**From fever of unknown origin to the inflammasome puzzle**.

The discovery of the macromolecular complex inflammasome resulted from observations made by investigators working with rare diseases (autoinflammatory syndromes), experts in apoptosis, and biochemists analyzing how cells sense the presence of danger. The first to identify the mechanism was a French consortium of scientists in 1997, which was due to the mutation causing familial Mediterranean fever (FMF). The mutated gene encoding an unusual structure of unknown function – named at that time marenostrin (from *mare nostrum*, referring to the Mediterranean Sea) – a pyrin involved in periodic fevers (French FMF Consortium, 1997). The protease which processes interleukin-1β [IL-1β-converting enzyme (ICE) = caspase-1] was identified in 1989 (Black et al., 1989) and cloned in 1992 (Cerretti et al., 1992; Thornberry et al., 1992). The identification of CARDIAK, a death domain associated with caspase-1, was identified in 1998 (Thome et al., 1998). The mutation in the gene (currently called NLRP3-inflammasome) in recurrent and chronic inflammation was initially detected in a group of rare autoinflammatory conditions, termed cryopyrin-associated periodic syndromes (CAPS) (Hoffman et al., 2001); this protein also belongs to the pyrin family. The link between pyrin, cryopyrin, NLRP3, death domain (CARD) and caspase-1 was established and this macromolecule complex was termed “inflammasome” (Martinon et al., 2002; Srinivasula et al., 2002) (**Figure 2**).

## IL-1 Receptors

The search for IL-1 receptors began in 1985 when a high-affinity plasma membrane receptor for human interleukin was identified (Dower et al., 1985); between 1985 and 1988, numerous investigators were working in this field of research (Bird and Saklatvala, 1986; Kilian et al., 1986; Kroggel et al., 1988).

The gene sequence for IL-1β and IL-1Ra receptors was described by Sims et al. (1988). Stylianou et al. (1992) was well established that the type I and not type II receptor led to signal transduction.

Eastgate et al. (1990) demonstrated the existence of an IL-1β binding protein, followed by the findings of Francesco Colotta and Alberto Mantovani in 1993 that the shed molecule of type II receptor was a decoy receptor and probably similar to the IL-1β-binding protein described by Eastgate and Duff.

In a recent book entitled “Body Messages: the Quest for the Proteins of Cellular Communication. Evolution of Message Discovery: The IL Family,” Fantuzzi (2016) elegantly describes the different facets, controversies and surprises characterizing the discovery of the IL-1, IL-1Ra and interleukin receptors families.

## Inflammasome, IL-1 and IL-Ra in Crystal-Induced Arthropathies

The first observation that IL-1 could contribute to the pathogenesis of crystal-induced arthritis Duff et al. (1983) demonstrated that monosodium urate (MSU) crystals were able to stimulate mononuclear phagocytes *in vitro* to produce EP. The authors failed to extend these results to other pathogenic crystals such as hydroxyapatite and calcium pyrophosphate (CPP), hypothesizing that the joint had to be, in some way, “conditioned” to propagate an inflammatory stimulus and that the source of IL-1 could lie outside the joint (Malawista et al., 1985). Subsequently, it became clear that purified crystals alone could not induce IL-1but that an additional stimulus was needed *in vitro* to generate IL-1 (Giamarellos-Bourboulis et al., 2009). Further *ex vivo* models identified free fatty acids (Joosten et al., 2010) and synovial fluid (SF) proteins (Scanu et al., 2016) as important triggers of IL-1 release.

An imbalance between the production of IL-1 and IL-1Ra induced by crystals in activated cells was pointed out in 1994 when it became clear that MSU and CPP crystals did not affect IL-1Ra production in neutrophils (Roberge et al., 1994).

Martinon et al. (2006) demonstrated for the first time the involvement of the inflammasome NLRP3 in caspase-1 activation and IL-1 release induced by crystals. IL-1R was also found to play a critical role in amplifying the inflammatory response through a MyD88-dependent mechanism (Chen et al., 2006). These observations, along with the discovery that uric acid was capable to stimulate the innate immune system (Shi et al., 2003), led to the addition of gout to the spectrum of autoinflammatory diseases (Punzi et al., 2012).

The key role that IL-1β plays in gout and pseudo-gout was further confirmed by the effectiveness of blocking agents to IL-1 in that it reduced acute attacks, initially observed after the administration of IL-1Ra anakinra, and then to the anti-IL-1β monoclonal antibody canakinumab (So et al., 2007; Schlesinger et al., 2012; Punzi and So, 2013). Accordingly, the recent EULAR recommendations for the management of gout advocate considering the administration of IL-1 blockers in those patients with frequent flares and contraindications to colchicine, NSAIDs and corticosteroids (Richette et al., 2017). In contrast, Rilonacept, a soluble receptor fusion protein binding both IL-1α and IL-1β, provided no benefit over indomethacin in an RCT (Terkeltaub et al., 2013). Other promising molecules, such as the recombinant human alpha-1-anti-trypsin (AAT)-IgG1 Fc fusion protein, have been shown to mainly target IL-1 in gout (Joosten et al., 2016).

## Effect of IL-1 on Cartilage and Bone in OA

The presence of a low molecular weight peptide released from synovial tissue and able to affect cartilage homeostasis was demonstrated at the end of the 1970s (Dingle et al., 1979). This messenger called “catabolin” was able to stimulate chondrocytes to degrade both cartilage proteoglycans and collagen (Saklatvala and Dingle, 1980), and soon its similarity to IL-1 was established (Saklatvala et al., 1984).

Gowen et al. (1983) demonstrated for the first time that IL-1-like factor altered the breakdown of bone by modulating both bone resorption in RA and bone formation in OA. As far as bone resorption is concerned, IL-1Ra was later shown to block the resorptive effect induced by IL-1 *in vitro* (Seckinger et al., 1990).

Despite the fact that the altered expression of proinflammatory cytokines and chemokines in OA cartilage and synovium is well documented (Kapoor et al., 2011; Raghu et al., 2016), the role of IL-1β has not been fully clarified. Very low levels of IL-1β have been determined in the synovial membrane and SF of patients with early and end-stage OA (Scanzello et al., 2009) although levels were more elevated in the SF cells of OA than in those of controls (Sauerschnig et al., 2014). However, IL-1β levels are significantly lower in OA than in RA (Farahat et al., 1993). *In vitro* studies have revealed that IL-1β plays a critical role in driving the production of proteolytic enzymes such as MMPs and ADAMTS-4 in OA (Bondeson et al., 2006). But results obtained from different OA animal models showed discrepancies when anti-IL-1β therapy (Fernandes et al., 1999; Zhang et al., 2004) or mice deficient in IL-1 (Clements et al., 2003) were utilized, thus emphasizing that other, IL-1-independent, pathways may be involved in OA pathology (Bondeson et al., 2010).

As far as the association between the IL-1 gene polymorphisms and OA is concerned, the haplotypes IL1A-IL1B-IL1RN and IL1B-IL1RN have, respectively, been shown to confer a higher and a decreased risk of OA (Smith et al., 2004). A study investigating the contribution of gene polymorphisms to dysregulated cytokine expression in OA cartilage revealed significant associations between the TNFα (high) chondrocyte phenotype and IL-1Ra allele 2, and between the TNFα (low) phenotype and IL-1β allele 2 (Moos et al., 2000).

An association between severe hand OA and single nucleotide polymorphisms in the IL-1R1 gene has been observed in family based and case-control analyses (Nakki et al., 2010). Several studies assessing the polymorphism in OA are ongoing, and some have been reviewed in a meta-analysis (Kerkhof et al., 2011).

## Toward Clinical Implications

Although IL-1 itself has been used as a therapeutic agent in oncology patients, alone it has little antitumor activity in melanoma, renal cell carcinoma, ovarian carcinoma or other diseases.

Considering that IL-1 seems to protect and restore the bone marrow from lesions induced by radiation or chemotherapy, it has been proposed to use it for treatment, but its modest hematopoietic effects probably do not counterbalance the toxicity necessary to achieve said effects (Veltri and Smith, 1996). IL-1α and IL-1β have also been proposed as vaccine adjuvants (Staats and Ennis, 1999).

The initial attempts to block IL-1 in infectious diseases have been unsuccessful and were accompanied by an increase in infections and side effects (Freeman and Buchman, 2001). Trials monitoring effects of IL-1Ra in patients with RA, which were begun in 1991, have produced interesting results although long-term results have not proved impressive and have demonstrated that this therapy is less effective than anti-TNF therapies. The initial trial targeting IL-1 in RA has been reviewed in detail (Dayer and Bresnihan, 2002).

At present, therapeutic blockade of IL-1 is crucial in many hereditary autoinflammatory diseases, as well as in patients with Still’s disease, crystal-induced arthropathies, certain skin diseases including neutrophil-triggered skin diseases, Behçet’s disease and deficiency of IL-1Ra, and other rare fever syndromes. Its effects have been marginal in RA patients and are still under debate in OA, type 2 diabetes mellitus, cardiovascular diseases and cancer (Larsen et al., 2009; Cantarini et al., 2015; Cavalli and Dinarello, 2015; Sfriso et al., 2016).

Relevant developments are still be expected in future with regard to the IL-1 family and the macromolecular complex which is inflammasome. The complexity of the various forms of the IL-1 receptor family, not discussed here in the history, is far from being fully understood and may lead to new avenues for pharmacological interventions.

## Note

We regret that given the brevity of the review with particular focus on rheumatological aspects, it was impossible to acknowledge all the important researchers involved in this field of study and their discoveries and contributions.

## Author Contributions

J-MD conceived and drafted the manuscript. FO and LP contributed to the drafting and the revision of the work. All authors approved it for publication.

## Conflict of Interest Statement

The authors declare that the research was conducted in the absence of any commercial or financial relationships that could be construed as a potential conflict of interest. The reviewer SJ and handling Editor declared their shared affiliation, and the handling Editor states that the process nevertheless met the standards of a fair and objective review.
